# Supplementary material for: Improved Catalytic Activity of Spherical Nucleic Acid Enzymes by Hybridization Chain Reaction and Its Application for Sensitive Analysis of Aflatoxin B1
Source: Sensors (Basel). 2024 Apr 5;24(7):2325. doi: 10.3390/s24072325 (PMC11014268; doi:10.3390/s24072325)
Supplement: Supplementary file 1 [file sensors-24-02325-s001.zip › sensors-2912118-supplementary.pdf]

Supporting Information

Improved Catalytic Activity of Spherical Nucleic Acid Enzymes by Hybridization Chain Reaction and its application for Sensitive Analysis of Aflatoxin B1

Wenjun Wang‡[1], Xuesong Li‡[1], Kun Zeng[1], Yanyan Lu[1], Boyuan Jia[1], Yibin Deng[2,3*], Jianxia Lv[4], Chenghao Wu[4], Xinyu Wang[1], Xinshuo Zhang[1], Zhen Zhang[1,2,3]*

[1]School of the Environment and Safety Engineering, Jiangsu University, Zhenjiang 212013, China.

[2]Center for Medical Laboratory Science, the Affiliated Hospital of Youjiang Medical University for Nationalities, Baise 533000, China.

[3]Key Laboratory of Clinical Molecular Diagnosis and Research for High Incidence Diseases in Western Guangxi, Guangxi, 533000, China.

[4] National Narcotics Laboratory Beijing Regional Center, Beijing 100164, China

*Corresponding author:

E-mail: zhangzhen@ujs.edu.cn

Fax: +86-511-88790955

‡ Both authors contributed equally to this paper.

Contents

Experimental Procedures 3

Sample preparation 3

Method for Calculating t-Test Statistics 3

Figures and Figure Captions 4

Figure S1. 4

Figure S2. 6

Figure S3. 7

Figure S4. 8

Figure S5. 9

Figure S7. 11

Figure S8. 12

Figure S9. 13

Figure S10. 14

Figure S11. 15

Tables and Table Captions 16

Table S1. 16

Table S2. 17

Table S3. 18

Table S4. 19

Table S5. 20

References 21

Experimental Procedures

Sample preparation

The five different kinds of food samples, including corn, red beans, rice, wheat, and peanuts, were purchased from a local supermarket. First, take appropriate amounts of samples and crush them with a grinder, and collect them through a 40-mesh sieve. Next, mix 5g of the sample with 25mL of methanol solution (volume ratio of methanol to water is 3 to 7) and shake for 10 minutes and then centrifuge the mixture at 4000 rpm for 8 minutes. Finally, the supernatant obtained is diluted 6 times with PBS buffer and used.

Method for Calculating t-Test Statistics

To assess the precision of two techniques, an assessment of the experimental outcomes was undertaken through a statistical comparison. The comparison entailed an initial F-test followed by an unpaired Student's t-test. For each sample, a statistical analysis was performed using a two-sample t-test, adopting equal sample sizes and equal variances. The process is described in detail below:

$$t = \frac{|\overline{x_1} - \overline{x_2}|}{S_{x1x2}} \sqrt{\frac{n}{2}} \qquad \text{(S1)}$$

$$S_{x1x2} = \sqrt{\frac{s_{x1}^2 + s_{x2}^2}{2}} \qquad \text{(S2)}$$

The symbols $\bar{x}$, Sx, and n indicate the average, standard deviation, and total number of parallel detections for the sample. In this equation, (S1) refers to the data acquired from the proposed method, where (S1) represents the data collected from the referenced method.

Figures and Figure Captions

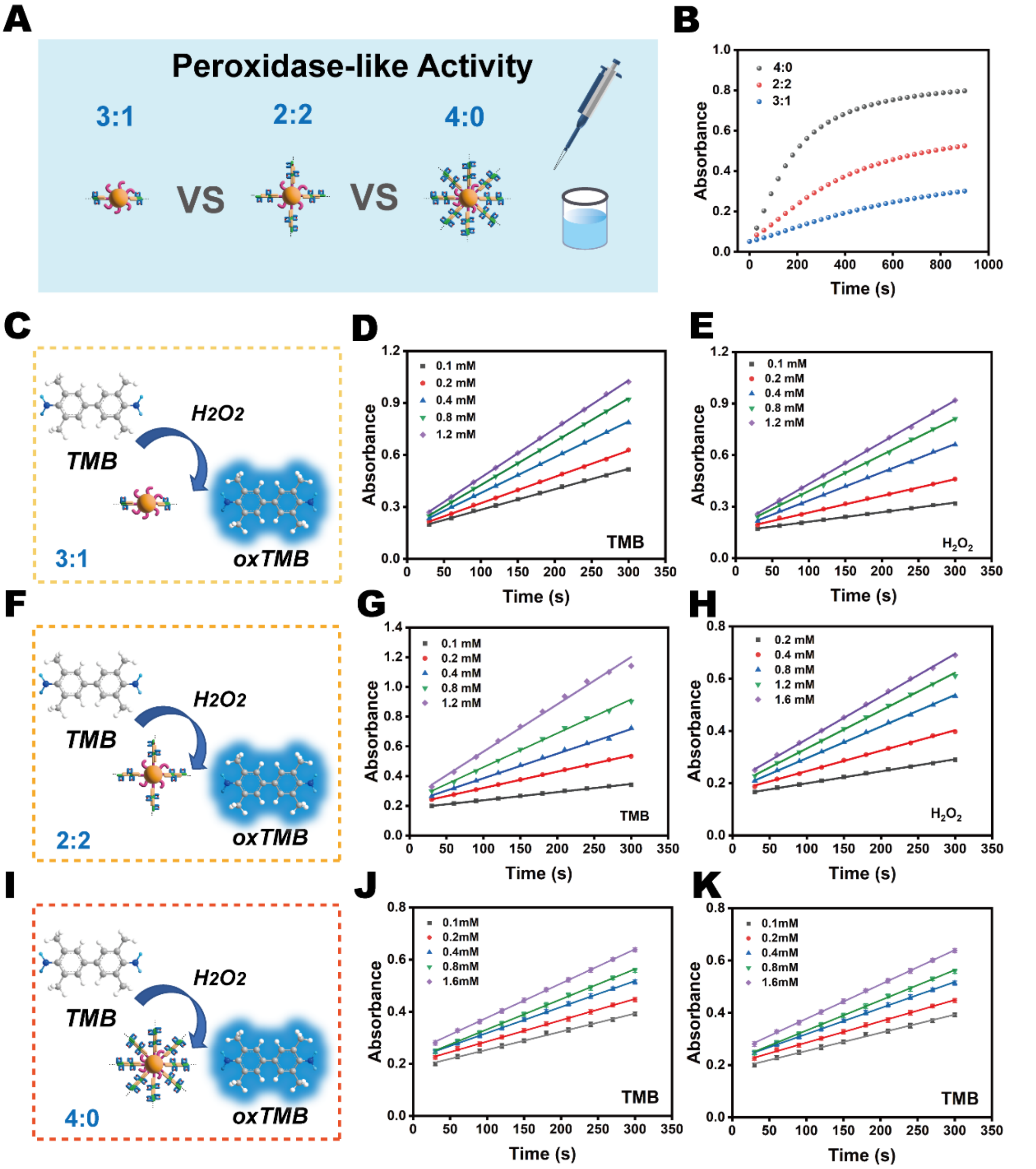

Figure S1. (A) Schematic diagram of the peroxidase-like activity assessment for 3:1, 2:2 and 4:0 split mode in TMB/H2O2 system. (B) Time-dependent absorbance curves in the presence of 3:1, 2:2 and 4:0 split mode respectively at 652 nm. (C) Schematic illustration of catalytic kinetics experiment for 3:1 split mode. (D) Curves between the time and absorbance toward different concentrations of TMB for 3:1 split mode. (E) Curves between the time and absorbance toward different concentrations of H2O2 for 3:1 split mode. (F) Schematic illustration of catalytic kinetics experiment for 2:2 split mode. (G) Curves between the time and absorbance toward different concentrations of

TMB for 2:2 split mode. (H) Curves between the time and absorbance toward different concentrations of H2O2 for 2:2 split mode. (I) Schematic illustration of catalytic kinetics experiment for 4:0 split mode. (J) Curves between the time and absorbance toward different concentrations of TMB for 4:0 split mode. (K) Curves between the time and absorbance toward different concentrations of H2O2 for 4:0 split mode.

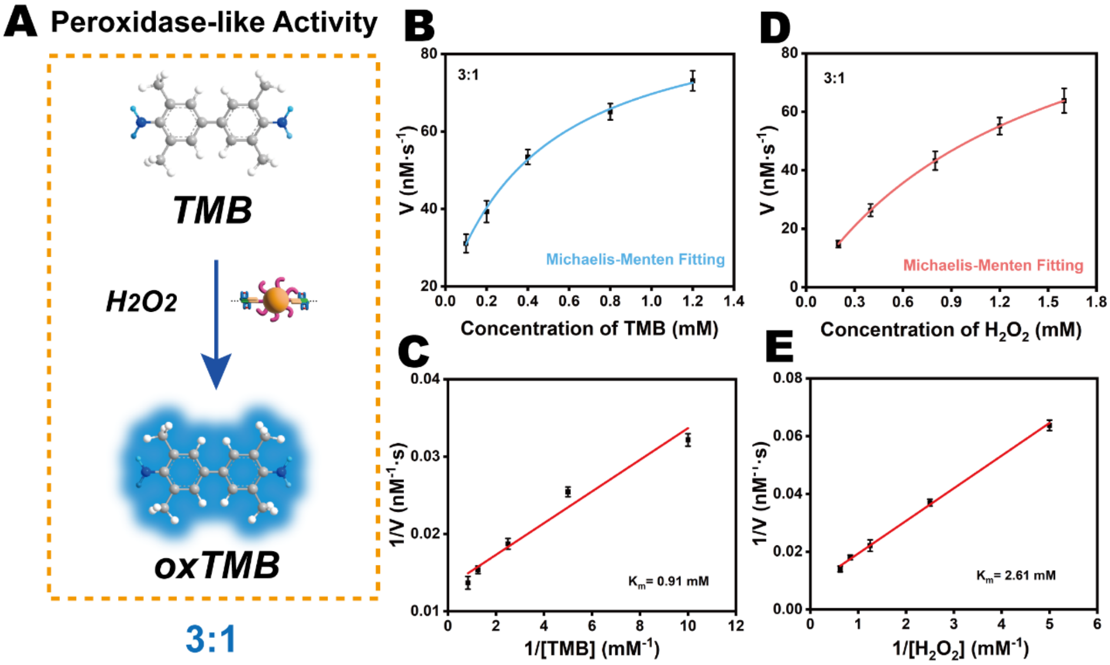

Figure S2. (A) Schematic illustrations of for catalytic kinetics assays of 3:1 split mode. (B) and (D) Steady-state kinetics curves of 3:1 split mode to TMB and H2O2 respectively. (C) and (E) Double-reciprocal plots for calculation of kinetic constants from the corresponding steady-state kinetics data.

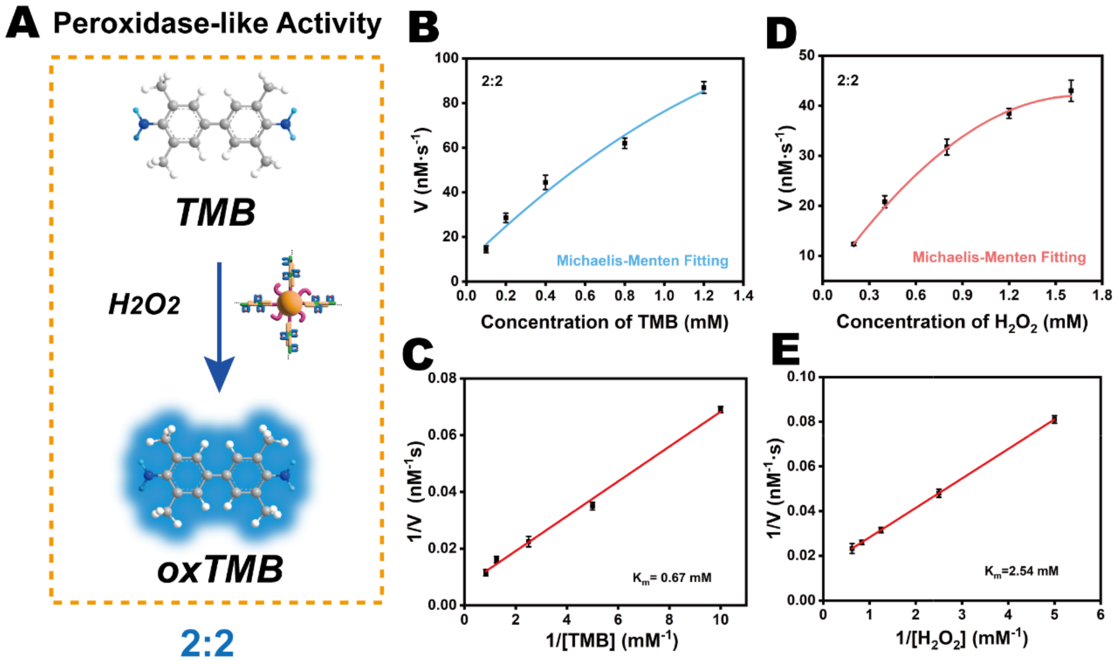

Figure S3. (A) Schematic illustrations of for catalytic kinetics assays of 2:2 split mode. (B) and (D) Steady-state kinetics curves of 2:2 split mode to TMB and H2O2 respectively. (C) and (E) Double-reciprocal plots for calculation of kinetic constants from the corresponding steady-state kinetics data.

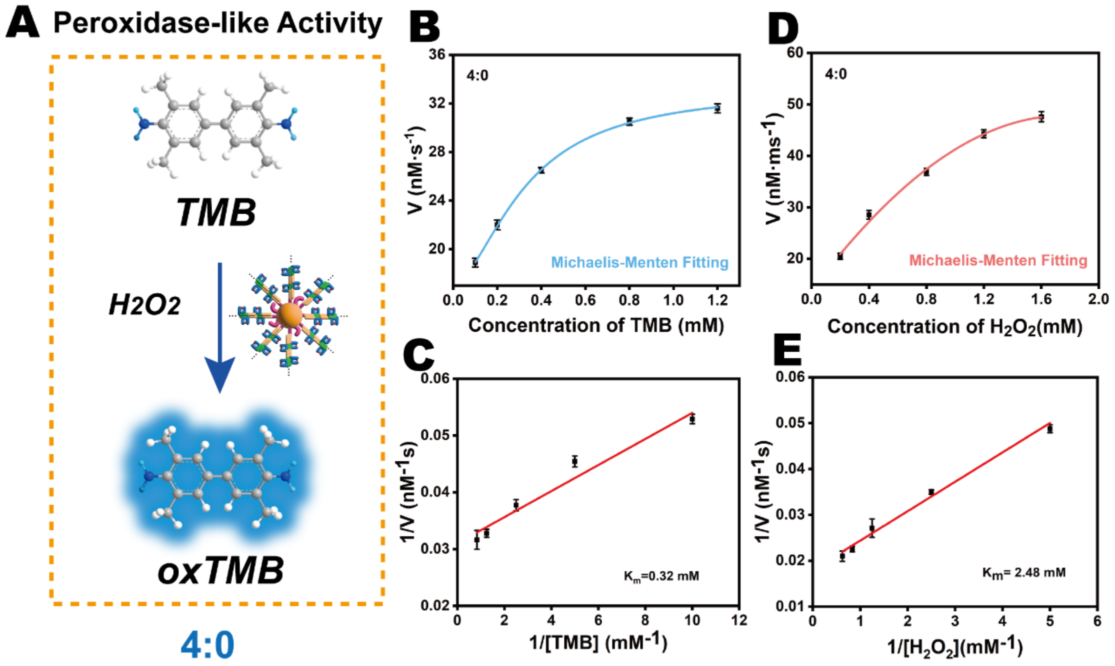

Figure S4. (A) Schematic illustrations of for catalytic kinetics assays of 4:0 split mode. (B) and (D) Steady-state kinetics curves of 4:0 split mode to TMB and H2O2 respectively. (C) and (E) Double-reciprocal plots for calculation of kinetic constants from the corresponding steady-state kinetics data.

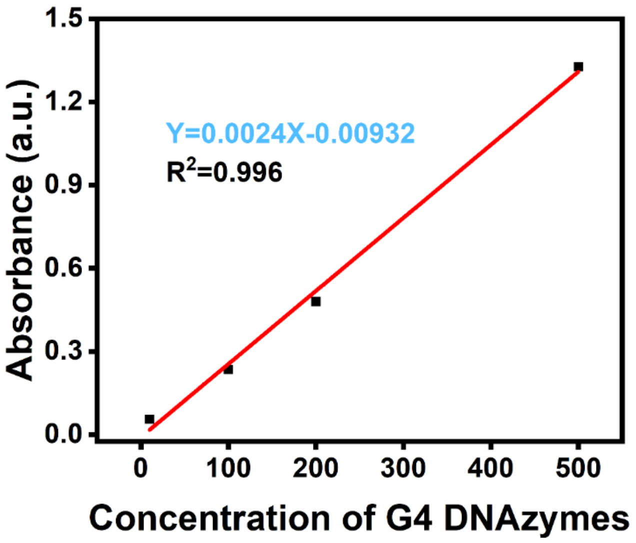

Figure S5. Linear standard curve of catalytic activity absorbance of G4 DNAzyme at different concentrations

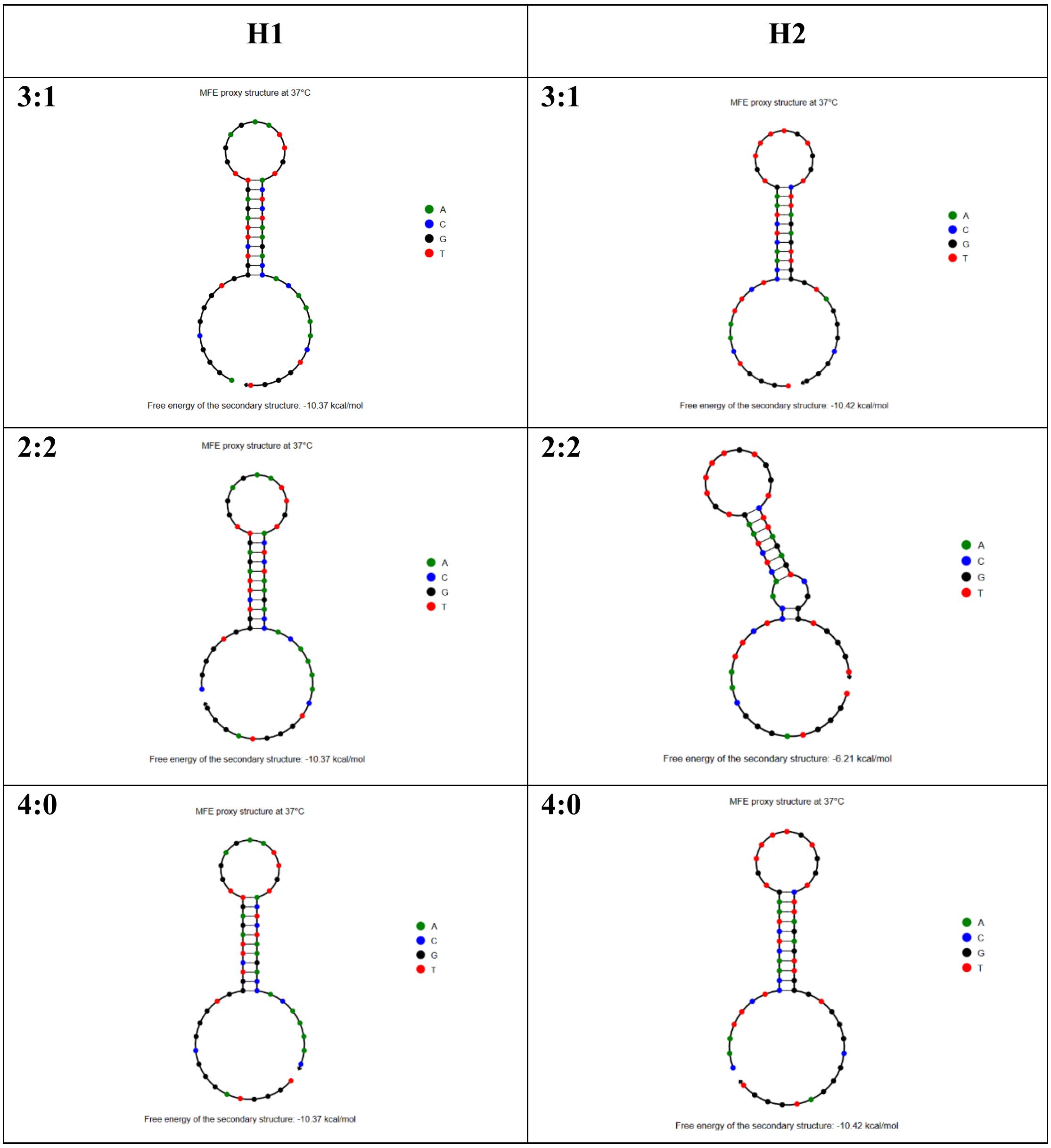

Figure S6. The secondary structure simulation results of hairpins with different split modes predicted from the from www.nupack.org. website.

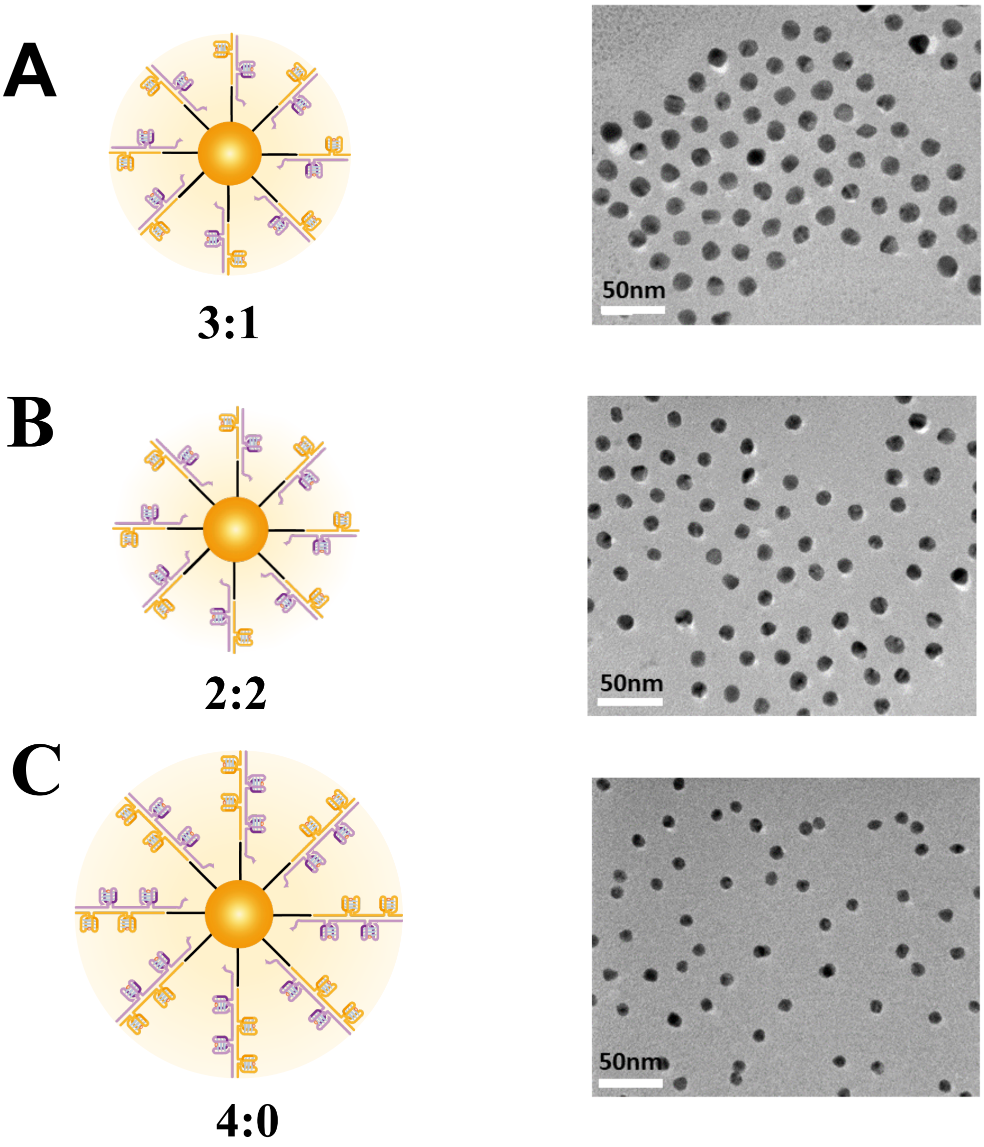

Figure S7. The TEM pictures of AuNPs-HCR with three different split modes. (A) 3:1 split mode; (B) 2:2 split mode; (C) 4:0 split mode.

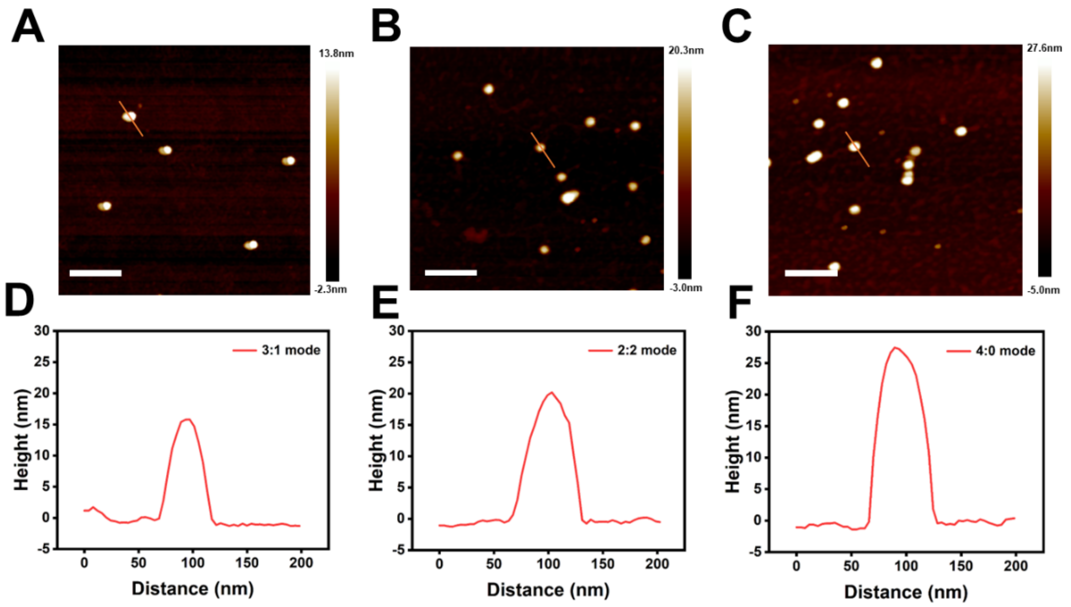

Figure S8. The AFM images and the corresponding height profiles of AuNPs-HCR. (A) 3:1 split mode; (B) 2:2 split mode; (C) 4:0 split mode.

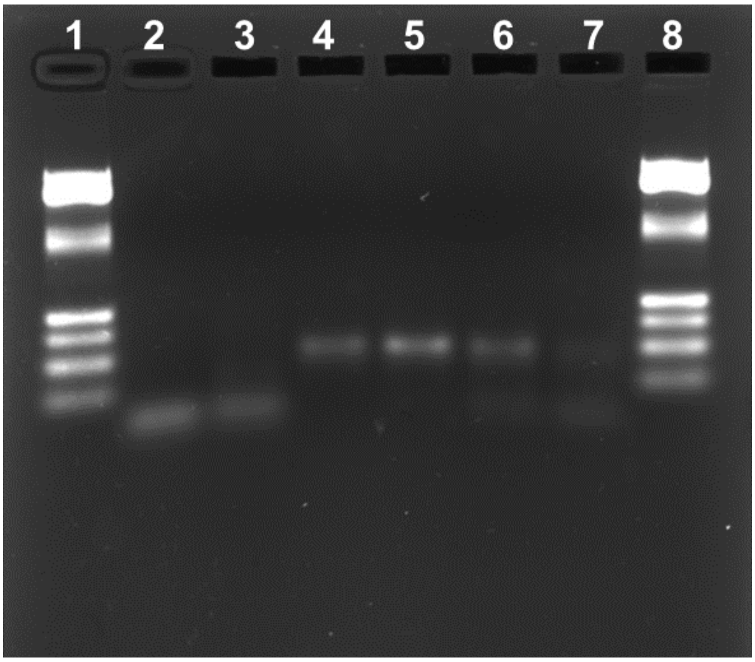

Figure S9. Agarose gel electrophoresis of the formation of a double-stranded structure in aptamer/S1. Lane 1: marker; Lane 2: Aptamer; Lane 3: S1; Lane 4: Aptamer and S1 double-stranded structure; Lane 5-7: Aptamer and S1 double-stranded structure with AFB1 at low, medium and high concentrations.

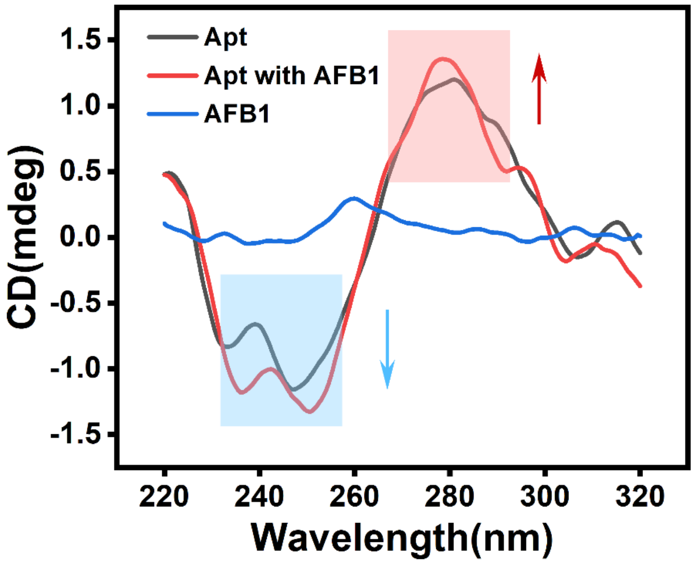

Figure S10. CD spectra of aptamer and AFB1 conjugate.

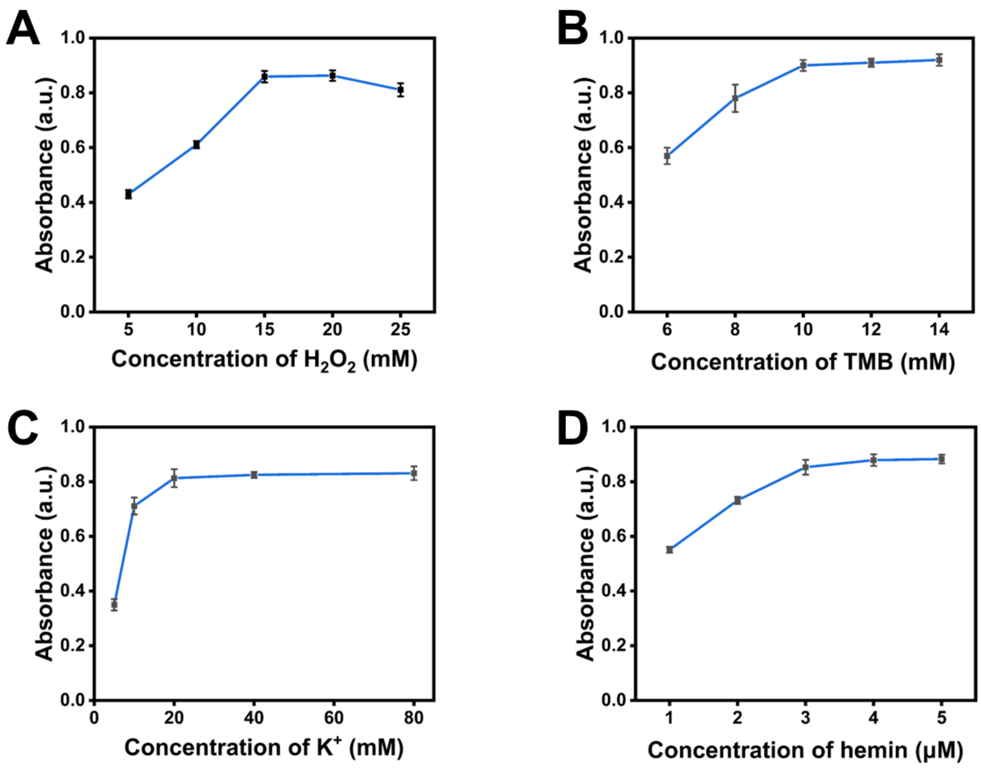

Figure S11. Optimization of this proposed method. (A-D) Optimization of the concentration of H_2O_2, TMB, K^+, and hemin.

Tables and Table Captions

Table S1. DNA Sequences.

Oligo	DNA sequence (5’-3’)
Apt-$_{AFB1}$	TGCACGTGTTGTCTCTCTGTGTCTCGTGC
S1	ACGAGACACAGAGAGACAACACGTG
I_A	CACGTGTTGTCTCGTTATAGCGATACTCTAA
I_B	GGGATATCTGTACCCACTAACTCTGTGTCTCGT
HP	SH-TTTTTTTTTTGGAGTTTTGTTTTTTGAGCGACACACTAT (rA)GGAAGAGATACTTTTTTCAAAACTCCA
Trigger	TTTTTTTTTTGGAGTTTTGTGGTCTTAGAGTAT
1:3 H1	AGGGCGGGTGGGTCTTAGAGTTGGAGAATTGTACTCTAAG ACCACAAAACTGGGT
1:3 H2	TGGGTCAATTCTCCAACTCTAAGTGTTTTGTGGTCTTAGAG TTGGGTAGGGCGGG
2:2 H1	CGGGTGGGTCTTAGAGTTGGAGAATTGTACTCTAAGACCA CAAAACTGGGTAGGG
2:2 H2	TGGGTAGGGCAATTCTCCAACTCTAAGTGTTTTGTGGTCTT AGAGTCGGGTGGGT
4:0 H1	TGGGTAGGGCGGGTGGGTCTTAGAGTTGGAGAATTGTACT CTAAGACCACAAAAC
4:0 H2	CAATTCTCCAACTCTAAGTGTTTTGTGGTCTTAGAGTTGGG TGGGCGGGATGGGT

DNA sequences are marked with different colors to better understand complementary pairing between different sequences and distinguish between different types of sequences. The blue represents the DNA tetranucleotide sequence; the green and light green represent the complementary parts of the hairpin; the red and dark red also represent the same meaning.

Table S2. Intra-assay and inter-assay variance of our proposed method for AFB1 detection.

AFB1 (ng/mL)	Intra-assay (n=6)		Inter-assay (n=3)	
	Mean ± SD (ng/mL)	RSD (%)	Mean ± SD (ng/mL)	RSD (%)
0.2	0.22 ±0.01	4.55	0.26 ± 0.02	7.69
0.5	0.53 ± 0.03	5.66	0.59 ± 0.05	8.47
1	1.11 ± 0.09	8.10	1.24 ± 0.07	5.64
2	2.17 ± 0.15	6.91	2.05 ± 0.12	5.85
5	5.27 ± 0.21	3.98	5.13 ± 0.17	3.31

Table S3. Recovery experiments of AFB1 in different real samples by this developed ELISA.

Samples	Background (ng/mL)	Spiked (ng/mL)	Found (ng/mL)	Recovery (%)	CV[a] (%)
Corn	ND	2.00	1.93	96.5	3.17
		5.00	4.89	97.8	4.53
		10.00	10.1	101	2.86
Red bean	ND	2.00	2.06	103	4.65
		5.00	5.08	101.6	3.22
		10.00	9.87	98.7	5.37
Rice	ND	2.00	2.06	103.0	3.84
		5.00	4.95	99	3.58
		10.00	9.76	97.6	3.41
Wheat	ND	2.00	1.95	97.5	2.67
		5.00	5.16	103.2	3.16
		10.00	9.67	96.7	2.73
Peanut	4.50	2.00	6.43	96.5	3.25
		5.00	9.87	107.4	5.31
		10.00	14.28	97.8	2.98

a: Coefficient variation (CV) was calculated by three parallel expermental dates.

Table S4. Comparison of the results from this work and commercial AFB1 ELISA Kit for contaminated naturally samples.

Type	Method conc. (mean ± SD, ng mL^{-1}, n=3)[a]		t_{exp}
	Our proposed method	Commercial ELISA Kit	
Corn	3.17 ± 0.12	3.49 ± 0.27	0.53
Red bean	3.72 ± 0.05	4.04 ± 0.27	0.46
Rice	1.18 ± 0.77	1.04 ± 0.23	0.46
Wheat	2.75 ±0.35	2.62 ± 0.42	0.33
Peanut	16.37± 0.02	15.98 ± 0.25	0.58

a: All samples of AFB1 exhibiting high concentrations were identified using a suitable dilution.

Table S5. The comparisons of our work with present methods for AFB1 determination.

Methods	Signal	LOD	Ref.
Fluorescence immunoassay	Fluorescent	0.21 ng/mL	[1]
Aptamer based sensor	Fluorescent	0.13 ng/mL	[2]
Photothermal immunoassay	Temperature	1.0 ng/mL	[3]
Bioluminescent immunoassay	Luminescent	0.05 ng/mL	[4]
Photothermal immunoassay	Photothermal	0.19 ng/mL	[5]
Microbalance immunoassay	Frequency	0.83 ng/mL	[6]
Colorimetric aptasensor	Colorimetric	0.18 ng/mL	[7]
Colorimetric biosensor	Colorimetric	10 ng/mL	[8]
This method	colorimetric	0.08 ng/mL	This work

References

1. Shu, Q.; Wu, Y.; Wang, L.; Fu, Z., A label-free immunoassay protocol for aflatoxin B1 based on UV-induced fluorescence enhancement. *Talanta* **2019,** *204*, 261-265.

2. Tan, H.; Ma, L.; Guo, T.; Zhou, H.; Chen, L.; Zhang, Y.; Dai, H.; Yu, Y., A novel fluorescence aptasensor based on mesoporous silica nanoparticles for selective and sensitive detection of aflatoxin B1. *Analytica Chimica Acta* **2019,** *1068*, 87-95.

3. Li, X.; Yang, L.; Men, C.; Xie, Y. F.; Liu, J. J.; Zou, H. Y.; Li, Y. F.; Zhan, L.; Huang, C. Z., Photothermal Soft Nanoballs Developed by Loading Plasmonic Cu2–xSe Nanocrystals into Liposomes for Photothermal Immunoassay of Aflatoxin B1. *Analytical Chemistry* **2019,** *91* (7), 4444-4450.

4. Ren, W.; Li, Z.; Xu, Y.; Wan, D.; Barnych, B.; Li, Y.; Tu, Z.; He, Q.; Fu, J.; Hammock, B. D., One-Step Ultrasensitive Bioluminescent Enzyme Immunoassay Based on Nanobody/Nanoluciferase Fusion for Detection of Aflatoxin B1 in Cereal. *Journal of Agricultural and Food Chemistry* **2019,** *67* (18), 5221-5229.

5. Li, X.; Yang, L.; Men, C.; Xie, Y. F.; Liu, J. J.; Zou, H. Y.; Li, Y. F.; Zhan, L.; Huang,C. Z. Photothermal Soft Nanoballs Developed by Loading Plasmonic Cu2–xSe Nanocrystals into Liposomes for Photothermal Immunoassay of Aflatoxin B1. *Anal. Chem.* **2019**, 91, 4444-4450.

6. Tang, Y.; Tang, D.; Zhang, J.; Tang, D., Novel quartz crystal microbalance immunodetection of aflatoxin B1 coupling cargo-encapsulated liposome with indicator-triggered displacement assay. *Analytica Chimica Acta* **2018,** *1031*, 161-168.

7. J. Lerdsri, W. Chananchana, J. Upan, T. Sridara, J. Jakmunee, Label-free colorimetric aptasensor for rapid detection of aflatoxin B1 by utilizing cationic perylene probe and localized surface plasmon resonance of gold nanoparticles. *Sens. Actuators B Chem. 320*, **2020**, 128356.

8. N. Hao, J. Lu, Z. Zhou, R. Hua, K. Wang, A pH-Resolved Colorimetric Biosensor for Simultaneous Multiple Target Detection, *ACS Sens.* **2018**, *3, 10,* 2159–2165.
